# Correlation between Time to Hyperbaric Oxygen Therapy and Delayed Neurological Sequelae in Acute Carbon Monoxide Poisoning Patients

**DOI:** 10.3390/diagnostics14020186

**Published:** 2024-01-15

**Authors:** Sungwoo Choi, Sangun Nah, Sangsoo Han

**Affiliations:** Department of Emergency Medicine, Soonchunhyang University Bucheon Hospital, Bucheon 14584, Republic of Korea; csw3613@naver.com (S.C.); potter325@naver.com (S.N.)

**Keywords:** carbon monoxide poisoning, hyperbaric oxygen therapy, time-to-treatment, decision tress, cognitive dysfunction

## Abstract

Carbon monoxide (CO) is one of the most common causes of intoxication. Delayed neurologic sequelae (DNS) have a major impact on prognosis of CO poisoning patients. Hyperbaric oxygen therapy (HBOT) is widely used to treat DNS. However, there is no consensus regarding the optimal timing of HBOT. This prospective study enrolled patients who visited the hospital from November 2019 to October 2022. The cutoff value for the latency to HBOT after CO exposure was determined, and the area under the receiver operating characteristic curve (AUC) was estimated. In total, 167 patients were divided into non-DNS and DNS groups. The initial Glasgow Coma Scale (GCS) score, CO exposure time, latency to HBOT after CO exposure, median length of hospital stay (*p* < 0.001) and creatine kinase (*p* = 0.016) showed significant differences. A GCS score ≤ 9 had an odds ratio (OR) of 5.059 (95% confidence interval [CI]: 1.602–15.976, *p* = 0.006), and latency to HBOT after CO exposure ≥ 200 min had an OR of 18.971 (95% CI: 4.310–83.508, *p* < 0.001). The AUC was 0.8235 (95% CI: 0.7504–0.8966). A GCS score ≤ 9 and latency to HBOT ≥ 200 min may be significant risk factors for DNS.

## 1. Introduction

Carbon monoxide (CO) is a colorless, tasteless, and odorless toxic gas produced by incomplete combustion of organic compounds [1]. Globally, the cumulative incidence and mortality of CO poisoning are 137 cases and 4.6 deaths per million, respectively [2]. CO exposure, which is one of the most common causes of poisoning, may occur in industrial sites, fire sites, and closed spaces without ventilation, and the number of cases of intentional CO poisoning as a means of suicide is increasing [3,4]. The primary toxicity of CO arises from its affinity for hemoglobin (Hb) in the body, which is >200 times higher than that for oxygen [5]. In addition, CO causes injury by affecting the oxidative process, myoglobin, and hepatic cytochromes, as well as the peroxidation of brain lipids [6]. These effects cause tissue hypoxia. The brain and heart are particularly vulnerable to ischemic injury, and they are thus prone to be damaged in CO poisoning patients [7,8]. Delayed neurologic sequelae (DNS), which may occur several days to several months after CO exposure, can manifest in personality changes, psychosis, cognitive impairment, or changes in consciousness [9,10,11]. Therefore, DNS is a critical factor in a patient’s long-term prognosis and quality of life, and prevention measures and appropriate treatment are very important [7].

In patients with CO poisoning, the half-life of carboxyhemoglobin (COHb) is approximately 320 min in room air, although this decreases to approximately 20 min when hyperbaric oxygen therapy (HBOT) is applied [12]. HBOT has been reported to be effective for preventing DNS and is the main treatment for CO poisoning [8,13,14]. Therefore, HBOT could be applied in patients with loss of consciousness, changes in personality, convulsions, neurologic deficit, suspected myocardial ischemia (chest pain, electrocardiogram changes, elevated cardiac markers), or a COHb level > 25% (>15% in pregnancy) [9,15]. According to some reports, applying HBOT as soon as possible after CO exposure is beneficial, and HBOT within 6 h can significantly reduce the severity of DNS [14,16,17]. Additionally, a recent study reported that applying HBOT within 22.5 h after CO exposure is effective [18]. However, there have been few studies on the impact of the timing of HBOT application on DNS. Additionally, the maximum pressure during HBOT sessions varies among studies from 2.5 to 3.0 atmospheres absolute (ATA). Previously, we performed HBOT with a maximum pressure of 3.0 ATA in a study that aimed to identify the optimal timing of HBOT to prevent DNS [13]. In this study, we further explored the relationship between DNS and the interval between CO exposure and HBOT application.

## 2. Materials and Methods

### 2.1. Study Design and Setting

This prospective, registry-based study was conducted in the emergency department (ED) of our tertiary care hospital in Gyeonggi-do, Republic of Korea, which has >65,000 patient visits per year. We enrolled all cases of CO poisoning in our registry, and all patient information was anonymized. The inclusion criteria were as follows: clear evidence of CO poisoning and a COHb level > 5% (in non-smokers; >10% in smokers) at the time of visiting the ED. This study was approved by our institutional review board (approval no. 2020-03-019).

This study targeted CO poisoning patients who visited our ED from November 2019 to October 2022. The exclusion criteria were as follows: aged <18 years, not undergoing HBOT, lack of data regarding CO exposure time, discharged against medical advice, persistent neurological symptoms at discharge, and loss to follow-up.

### 2.2. Study Variables and Definitions

Variables that may be associated with the prognosis of acute CO poisoning in patients were investigated in this study. Clinical characteristics of interest in the CO registry included age, sex, body mass index, vital signs on arrival at the ED, comorbidities (hypertension and diabetes mellitus), smoking status, intentionality, Glasgow Coma Scale [GCS] score ≤ 9 [19], CO exposure time, interval between HBOT and arrival at the ED, interval between HBOT and exposure, symptoms (headache, loss of consciousness, chest pain, and dyspnea), laboratory findings (COHb, white blood cell count, blood urea nitrogen level, creatinine level, creatine kinase level, arterial pH, and C-reactive protein, lactate, and troponin I levels), and length of hospital stay.

DNS was defined as the onset of any neurological sign or symptom within 3 months of discharge from the hospital (e.g., cognitive decline, motor deficits, dysarthria, dysphagia, Parkinsonism, dyspraxia, psychosis, depression, or seizures) [20]. If the patient was suspected of DNS based on telephone interviews or their behavior in the outpatient clinic or ED, they were asked to return to the hospital and consult with a neurologist. New-onset or worsening depression after CO poisoning was confirmed through an interview with a psychiatrist. After excluding other possible causes, DNS was ultimately diagnosed by the neurologist.

### 2.3. HBOT Protocol

In our institute, all CO poisoning patients are immediately treated with normobaric oxygen therapy, and, if the patient meets the indications, HBOT is performed. The indications for HBOT in our hospital are as follows: COHb level ≥ 25%, myocardial ischemia suspected on the basis of elevated cardiac enzymes or chest pain, and neurologic deficits, such as loss of consciousness, impaired cognitive function, altered mental state, or seizure. According to our HBOT protocol, three sessions of HBOT were performed within 24 h: the first session was performed for 150 min at 3 ATA, and the other sessions were performed for 120 min at 2 ATA [13].

### 2.4. Statistical Analysis

The data of the non-DNS and DNS groups were analyzed using the Mann–Whitney U test, *t*-test (continuous variables), Fisher’s exact test, or χ^2^ test (categorical variables). On the basis of Youden’s index (sensitivity + specificity − 1), the optimal cutoff value for the interval between HBOT and CO to distinguish between the two groups was determined. Stepwise multiple logistic regression analysis was used to identify risk factors for DNS, and the results are presented as odds ratios (ORs) with 95% confidence intervals (CIs). Multicollinearity was estimated according to variance inflation factor (VIF) values. Variables with VIF values < 5 are considered to have no effect on the results of regression analyses, while those with values > 10 are considered to have a significant effect on the results [21]. To assess model performance, the area under the receiver operating characteristic curve (AUC) was estimated. We also constructed a decision tree based on the results of the multivariable regression analysis to obtain prediction algorithms [22]. Two-tailed *p*-values < 0.05 were considered indicative of statistical significance. R (version 4.2.2; R Foundation for Statistical Computing, Vienna, Austria) and SPSS (version 26.0; IBM Corp., Armonk, NY, USA) software were used for the statistical analysis.

## 3. Results

**Figure 1:**
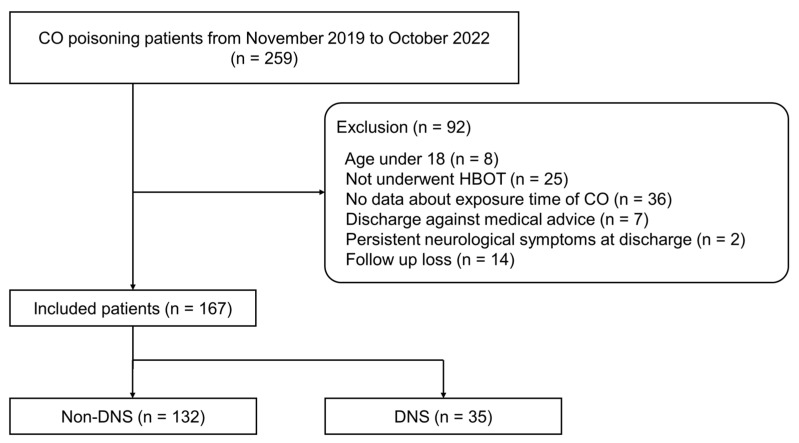
Flow chart of patient selection. Abbreviations: CO, carbon monoxide; HBOT, hyperbaric oxygen therapy; DNS, delayed neurologic sequelae.

A total of 259 CO poisoning patients visited our ED during the study period. Of these patients, 92 were excluded for the following reasons: aged <18 years (*n* = 8), did not undergo HBOT (*n* = 25), lack of data about CO exposure time (*n* = 36), discharged against medical advice (*n* = 7), persistent neurological symptoms at discharge (*n* = 2), and lost to follow-up (*n* = 14). Thus, 167 patients were divided into non-DNS (*n* = 132, 79%) and DNS (*n* = 35, 21%) groups and analyzed (Figure 1).

### 3.1. Baseline Clinical Characteristics

The baseline clinical characteristics of the patients are summarized in Table 1. The median age of the patients was 42 years, and 117 (70.1%) patients were male. There were 18 patients (10.8%) with hypertension, 13 (7.8%) with diabetes mellitus, and 78 (46.7%) current smokers. The median GCS score at the time of arrival at the ED was 15. In total, 125 patients (74.9%) intentionally exposed themselves to CO. The median exposure time was 99 min, and DNS developed in 35 patients (21%).

### 3.2. Clinical Characteristics according to DNS Status

The non-DNS and DNS groups differed significantly in terms of the GCS score at presentation (15 vs. 13; *p* < 0.001), CO exposure time (90 vs. 144 min; *p* < 0.001), interval between HBOT and CO exposure (197.5 vs. 281 min; *p* < 0.001), creatine kinase level (121 vs. 203 U/L; *p* = 0.016), and median length of hospital stay (3 vs. 5 days; *p* < 0.001) (Table 2).

### 3.3. Risk Factors for DNS in Cases of Acute CO Poisoning

**Figure 2:**
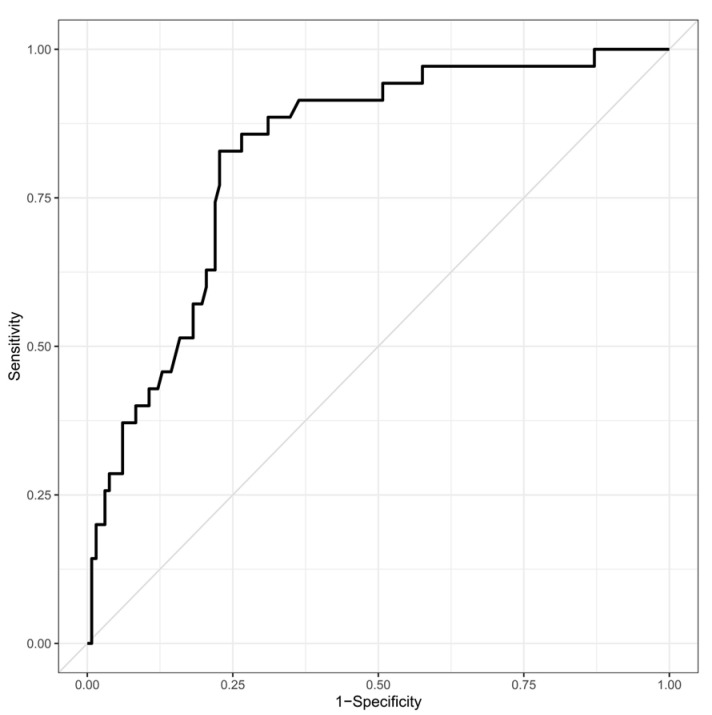
Receiver operating characteristic curve for the multivariable logistic regression model (area under the curve = 0.8235, 95% confidence interval: 0.7504–0.8966).

The initial GCS score and interval between HBOT and CO exposure were analyzed by multiple logistic regression to identify risk factors for DNS in acute CO poisoning patients. A GCS score ≤ 9 (OR = 5.059, 95% CI: 1.602–15.976, *p* = 0.006) and interval between HBOT and CO exposure ≥ 200 min (OR = 18.971, 95% CI: 4.310–83.508, *p* < 0.001) were significant risk factors for DNS. The independent variables did not exhibit multicollinearity, i.e., all VIF values were <5 (Table 3). The AUC of the multiple logistic regression model was 0.8235 (95% CI: 0.7504–0.8966) (Figure 2).

**Figure 3:**
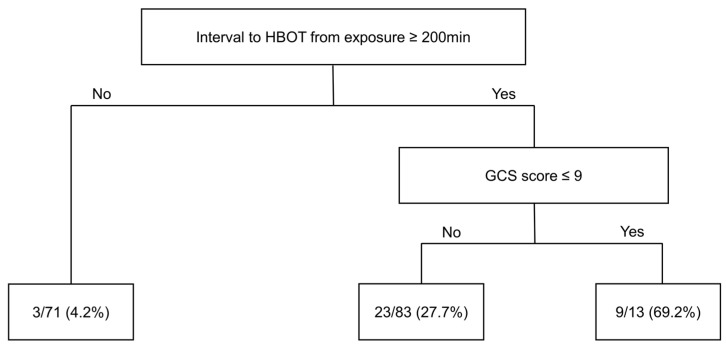
Decision tree for the 167 patients. First, patients were stratified according to the interval between HBOT and CO exposure, with the threshold set at 200 min. Then, patients above the threshold were stratified according to the GCS score (threshold = 9 points). Percentages in the bottom box indicate the DNS occurrence rate. Abbreviations: HBOT, hyperbaric oxygen therapy; GCS, Glasgow Coma Scale; DNS, delayed neurologic sequelae.

The decision tree included all 167 patients enrolled in our study. First, patients were grouped according to the interval between HBOT and CO, taking 200 min as the threshold. The proportion of DNS among patients for whom the interval was below the threshold was 4.2%. The 96 patients for whom the interval was equal to or above the threshold were further stratified into two subgroups based on a GCS score threshold of 9 points; patients for whom the GCS score was equal to or above the threshold had a DNS rate of 69.2%, while those under the threshold had a DNS rate of 27.7% (Figure 3).

## 4. Discussion

In this study, a GCS score < 9 at the time of arrival at the ED and an interval between CO exposure and HBOT ≥ 200 min were confirmed as significant risk factors for DNS.

DNS can occur at an early stage or up to several months after CO exposure. Careful attention and observation are required because various neurological symptoms can arise from CO exposure [9,10,11]. Changes in consciousness or neurological symptoms immediately after CO exposure are caused by ischemic injury to the brain, where CO has a high affinity for Hb [13,18]. Excessive dopamine, an increase in reactive oxygen species, impaired antioxidant action, lipid peroxidation, cytochrome expression, and NMDA receptor activation are putative causes of DNS [7,23,24]. However, the pathophysiology of DNS has not been fully elucidated. Currently, HBOT is the main treatment for DNS [3,13,18,25]. In addition, drugs inducing hypothermia, neuroprotective drugs, oxidative stress inhibitors, drugs inducing apoptosis, and oxidative stress inhibitors show promise but are still in the animal testing stage [18,26].

HBOT increases dissolved oxygen in the blood and accelerates the removal of CO [13,27]. It also effectively prevents lipid peroxidation in the brain and preserves ATP in tissues [13]. In animal models, HBOT has been reported to exert beneficial effects by inhibiting leukocyte beta-2 integrins, reversing CO–cytochrome c oxidase binding, restoring energy metabolism, and reducing oxidative stress [28,29,30,31]. However, HBOT can itself cause oxidative stress, depending on the pressure and exposure time [32,33]. It has been reported that HBOT at 2.0 ATA does not provide much benefit, and the results are worse when two rather than one session are completed [34,35]. However, HBOT can prevent DNS when carried out at 2.5–2.8 or 2.5–3.0 ATA [13,14].

In addition, it has been reported that HBOT should be applied as soon as possible when a patient meets the indications. However, no consensus has been reached regarding the optimal timing of HBOT [14,16,17,18]. Lee et al. distinguished early (within 6 h) and late (6–24 h) groups according to the time between CO exposure and HBOT, and the early group showed better neurological outcomes [17]. Liao et al. suggested an optimal cutoff for the latency to HBOT after CO exposure of 22.5 h based on Youden’s index [18]. However, in this study, the risk of DNS significantly increased when the interval between HBOT and CO exposure was ≥200 min. Previous studies showed that the rate of intentional CO poisoning for the purpose of suicide was only 30–40%. However, in this study, intentional poisoning accounted for about 74% of all cases.

It has been reported that intentional CO poisoning for the purpose of suicide has a worse prognosis [36]. Because this study had a large proportion of such patients, the optimal cutoff time was shorter than that of other studies. Our results suggest that HBOT should be applied as soon as possible, and the cutoff should thus be more stringent than those suggested in previous studies. For patients who meet the indications, performing HBOT as soon as possible could prevent DNS.

Our study confirmed that the risk of DNS increases when the GCS score is <9 points, in line with previous studies [9,18,37]. A low initial GCS score may delay the discovery of DNS, prolonging not only the CO exposure time but also the interval between arrival at the hospital and HBOT, ultimately resulting in more severe poisoning [18]. The more prolonged the CO exposure, the more likely it is that neurological symptoms or decreased consciousness will occur, which can also increase the likelihood of DNS [38]. Therefore, accurately determining the initial GCS score is important for a patient’s prognosis and should inform the treatment applied.

In this study, a decision tree was constructed to determine the risk of DNS according to the interval from CO exposure to HBOT and the GCS score. The decision tree can also be used as a supplementary tool to predict a patient’s prognosis. However, this study also had several limitations. First, we diagnosed DNS based on clinical symptoms and interviews. A standardized tool has not yet been developed to diagnose DNS, and we diagnosed it using criteria presented in a previous study [20]. Second, most of our participants had intentionally exposed themselves to CO for the purpose of suicide. Because these participants may also have ingested other drugs or ethanol, our ability to interpret their GCS scores solely in terms of CO exposure was limited. Third, among the patients enrolled in our study, only those who met the indications for HBOT were analyzed. Therefore, it is difficult to generalize the results to all CO patients. However, since it is known that individuals who are not indicated for HBOT have a low risk of developing DNS, the results of this study can be considered meaningful [39]. Finally, this was a single-center study; the HBOT protocol may differ among centers, and the racial and regional characteristics of patients may also influence outcomes. Therefore, it is difficult to generalize the results to other regions or countries, and prospective multicenter studies are needed to validate our findings.

## 5. Conclusions

This study confirmed associations of DNS with the initial GCS score and the interval between CO exposure and HBOT in CO poisoning patients. More specifically, a GCS score ≤ 9 and a latency to HBOT ≥ 200 min were significant risk factors for DNS. If a patient is indicated for HBOT, it should be performed as soon as possible to prevent DNS.

## Figures and Tables

**Table 1:** General characteristics of the study population.

	Total (*N* = 167)
Age, years	42 [31–52.5]
Males, *n* (%)	117 (70.1)
BMI, kg/m^2^	24.0 [21.3–26.3]
Comorbidities, *n* (%)	
Hypertension	18 (10.8)
Diabetes mellitus	13 (7.8)
Current smoker, *n* (%)	78 (46.7)
Initial GCS score	15 [13–15]
Intentionality (%)	125 (74.9)
CO exposure time, min	99 [50, 150]
Interval between HBOT and exposure, min	239 [145.5–300]
Length of hospital stay, days	3 [2–4]
Rate of DNS (%)	35 (21.0)

Note: Values are expressed as median [interquartile range] or number (proportion). Abbreviations: BMI, body mass index; GCS, Glasgow Coma Scale; CO, carbon monoxide; HBOT, hyperbaric oxygen therapy; DNS, delayed neurologic sequelae.

**Table 2:** Comparison of baseline characteristics between the non-DNS and DNS groups.

	Non-DNS	DNS	*p*-Value
	(*n* = 132)	(*n* = 35)	
Age, years	41 [30–52.3]	44 [37–52.5]	0.376
Male, *n* (%)	94 (71.2)	23 (65.7)	0.672 *
BMI, kg/m^2^	23.9 [21.3–26.2]	24 [21.3–26.6]	0.594
Initial GCS score	15 [14–15]	13 [9–15]	<0.001
Initial GCS score ≤ 9, *n* (%)	9 (6.8)	10 (28.6)	0.001 **
Vital signs			
Systolic blood pressure, mmHg	130 [120–140]	130 [111–145]	0.893
Diastolic blood pressure, mmHg	80 [73–90]	80 [70–94.5]	0.986
Heart rate, beats/min	90 [80–102.3]	90 [78–100]	0.795
Respiratory rate, breaths/min	20 [18.8–20]	20 [18–20]	0.208
Body temperature, °C	36.8 [36.5–37.1]	36.8 [36.5–37.3]	0.833
Oxygen saturation, %	98 [96–99]	98 [96–99]	0.704
Comorbidities, *n* (%)			
Hypertension	11 (8.3)	7 (20)	0.064 **
Diabetes mellitus	11 (8.3)	2 (5.7)	>0.99 **
Current smoker, n (%)	63 (47.7)	15 (42.9)	0.747 *
Exposure time of CO, min	90 [44.5, 126.3]	144 [89, 229.5]	<0.001
Interval to HBOT from exposure, min	197.5 [132.3–295.3]	281 [241.5–370]	<0.001
Intentionality, *n* (%)	96 (72.7)	29 (82.9)	0.313 *
Symptoms, *n* (%)			
Headache	13 (9.9)	3 (8.6)	>0.99 **
Loss of consciousness	35 (26.5)	9 (25.7)	>0.99 *
Dyspnea	11 (8.3)	1 (2.9)	0.463 **
Chest pain	5 (3.8)	1 (2.9)	>0.99 **
Laboratory findings			
COHb, %	12.3 [6.2–19.0]	12.7 [11.3–17.6]	0.160
White blood cells, ×10^3^/mm^3^	11.4 [8.2–14.9]	11.6 [8.3–16.4]	0.803
Blood urea nitrogen, mg/dL	12.6 [10.8–16.6]	14.3 [11.3–21.0]	0.137
Creatinine, mg/dL	1 [0.8–1.1]	1 [0.9–1.2]	0.399
Creatine kinase, U/L	121 [83.8–220.3]	203 [100–1,199.5]	0.016
Arterial pH	7.41 [7.37–7.43]	7.41 [7.38–7.44]	0.969
C-reactive protein, mg/dL	0.11 [0.04–0.34]	0.2 [0.09–0.6]	0.050
Lactate, mmol/L	1.9 [1.5–2.4]	2.1 [1.5–2.4]	0.534
Troponin I, ng/mL	0.1 [0.1–0.24]	0.1 [0.1–−0.63]	0.615
Length of hospital stay, days	3 [2–3]	5 [3–11]	<0.001

Note: Values are expressed as median [interquartile range] or number (proportion). * Pearson’s χ^2^ test; ** Fisher’s exact test. Abbreviations: DNS, delayed neurologic sequelae; BMI, body mass index; GCS, Glasgow Coma Scale; CO, carbon monoxide; HBOT, hyperbaric oxygen therapy; ED, emergency department; COHb, carboxyhemoglobin.

**Table 3:** Multivariable logistic regression analysis of risk factors for delayed neurologic sequelae in patients with acute CO poisoning.

	OR (95% CI)	*p*-Value	VIF
Initial GCS score ≤ 9	5.059 (1.602–15.976)	0.006	1.046
Interval between HBOT and CO exposure ≥ 200 min	18.971 (4.310–83.508)	<0.001	1.307
COHb, %	1.043 (0.995–1.093)	0.079	1.323

Note: Values are expressed as median [interquartile range] or number (proportion). Abbreviations: BMI, body mass index; GCS, Glasgow Coma Scale; CO, carbon monoxide; HBOT, hyperbaric oxygen therapy; DNS, delayed neurologic sequelae.

## Data Availability

Data are available from the author (Sangsoo Han; brayden0819@daum.net) of this publication.
